# Engagement of cellular prion protein with the co-chaperone Hsp70/90 organizing protein regulates the proliferation of glioblastoma stem-like cells

**DOI:** 10.1186/s13287-017-0518-1

**Published:** 2017-04-17

**Authors:** Rebeca Piatniczka Iglesia, Mariana Brandão Prado, Lilian Cruz, Vilma Regina Martins, Tiago Góss Santos, Marilene Hohmuth Lopes

**Affiliations:** 1 Laboratory of Neurobiology and Stem cells, Department of Cell and Developmental Biology; Institute of Biomedical Sciences, University of Sao Paulo, Av. Prof. Lineu Prestes, 1524 - Cidade Universitária “Armando Salles Oliveira”, Butanta - Sao Paulo, SP 05508-000 Brazil; 2 Laboratory of Cell and Molecular Biology, International Research Center, A.C. Camargo Cancer Center, Sao Paulo, SP 02056-070 Brazil

**Keywords:** Cellular prion protein, Hsp70/90 organizing protein, Glioblastoma, Stem cells, Proliferation

## Abstract

**Background:**

Glioblastoma (GBM), a highly aggressive brain tumor, contains a subpopulation of glioblastoma stem-like cells (GSCs) that play roles in tumor maintenance, invasion, and therapeutic resistance. GSCs are therefore a promising target for GBM treatment. Our group identified the cellular prion protein (PrP^C^) and its partner, the co-chaperone Hsp70/90 organizing protein (HOP), as potential target candidates due to their role in GBM tumorigenesis and in neural stem cell maintenance.

**Methods:**

GSCs expressing different levels of PrP^C^ were cultured as neurospheres with growth factors, and characterized with stem cells markers and adhesion molecules markers through immunofluorescence and flow cytometry. We than evaluated GSC self-renewal and proliferation by clonal density assays and BrdU incorporation, respectively, in front of recombinant HOP treatment, combined or not with a HOP peptide which mimics the PrP^C^ binding site. Stable silencing of HOP was also performed in parental and/or PrP^C^-depleted cell populations, and proliferation in vitro and tumor growth in vivo were evaluated. Migration assays were performed on laminin-1 pre-coated glass.

**Results:**

We observed that, when GBM cells are cultured as neurospheres, they express specific stemness markers such as CD133, CD15, Oct4, and SOX2; PrP^C^ is upregulated compared to monolayer culture and co-localizes with CD133. PrP^C^ silencing downregulates the expression of molecules associated with cancer stem cells, upregulates markers of cell differentiation and affects GSC self-renewal, pointing to a pivotal role for PrP^C^ in the maintenance of GSCs. Exogenous HOP treatment increases proliferation and self-renewal of GSCs in a PrP^C^-dependent manner while HOP knockdown disturbs the proliferation process. In vivo, PrP^C^ and/or HOP knockdown potently inhibits the growth of subcutaneously implanted glioblastoma cells. In addition, disruption of the PrP^C^-HOP complex by a HOP peptide, which mimics the PrP^C^ binding site, affects GSC self-renewal and proliferation indicating that the HOP-PrP^C^ complex is required for GSC stemness. Furthermore, PrP^C^-depleted GSCs downregulate cell adhesion-related proteins and impair cell migration indicating a putative role for PrP^C^ in the cell surface stability of cell adhesion molecules and GBM cell invasiveness, respectively.

**Conclusions:**

In conclusion, our results show that the modulation of HOP-PrP^C^ engagement or the decrease of PrP^C^ and HOP expression may represent a potential therapeutic intervention in GBM, regulating glioblastoma stem-like cell self-renewal, proliferation, and migration.

**Electronic supplementary material:**

The online version of this article (doi:10.1186/s13287-017-0518-1) contains supplementary material, which is available to authorized users.

## Background

Glioblastoma (GBM) is the most common and aggressive type of central nervous system (CNS) tumor and is one of the most lethal human cancers [1]. This aggressive behavior has been attributed to a highly proliferative subset of cells called glioblastoma stem-like cells (GSCs) that contribute to tumor initiation and therapeutic resistance [2]. These cells are thought to be responsible for maintaining GBM tumors after therapy and repopulating them after total resection [3]. In addition, they are involved in tumor angiogenesis [4] and immune evasion [5], promoting tumor progression.

The cellular prion protein (PrP^C^) and the Hsp70/Hsp90 organizing protein (HOP) modulate several stem cell functions. PrP^C^ regulates proliferation and self-renewal of neural stem cells (NSCs) [6], while HOP participates in the maintenance of pluripotent stem cells [7]. Previous data from our group show that PrP^C^ modulates NSC proliferation and self-renewal through its interaction with HOP [6]. Both PrP^C^ and HOP also modulate tumorigenesis, affecting the progression and maintenance of different types of cancers [8]. PrP^C^ associates with a poor clinical outcome and survival in pancreatic ductal adenocarcinoma and melanoma [9, 10], and with invasion and metastasis in gastric and breast cancers [8, 11, 12]. Depleting PrP^C^ inhibits growth, promotes programmed cell death in gliomas [13], and sensitizes tumor cells to cytotoxic drugs [14]. Likewise, HOP expression correlates with tumor progression [15], and is associated with proliferation [16, 17], invasion [17], and poor patient prognosis [18], and HOP has been described as an important regulator of tumor maintenance [19–21]. Recent findings show that HOP-PrP^C^ binding modulates migration and invasion of colorectal cancer cells [22]. Erlich et al. [16] reported that the interaction of PrP^C^ with HOP modulates proliferation in glioma cell lines, and data from our group showed that higher expression of both proteins is correlated with greater tumor proliferation and lower survival in patients with GBM [15]. In addition, blocking the HOP-PrP^C^ complex decreases tumor growth and increases survival in an animal model [15], making it a potential target for GBM therapy.

While the HOP-PrP^C^ complex participates both in GBM tumorigenesis and in the maintenance of NSCs—which are believed to originate GSCs—their role in GSC biology is still unclear. Therefore, this study aimed to elucidate the role of the HOP-PrP^C^ complex in the regulation of GSCs by downregulating the expression of PrP^C^ in a human GBM cell line cultured as GSCs and by testing how proliferation and self-renewal properties were affected in the presence of exogenous HOP; we also wished to evaluate the therapeutic potential of targeting this complex using synthetic peptides in attempt to alter GSC biology. Additionally, we investigated whether PrP^C^ may be used as a novel biomarker for GBM by studying its role in GSC stemness.

## Methods

### Proteins and peptides

Mouse recombinant HOP was purified as previously described [23]. Human pepHOP_230–245_ (ELGNDAYKKKDFDTAL) and C-terminal pepHOP_422–437_ (GCKTVDLKPDWGKGYS) peptides were synthesized by GenScript (Piscataway, NJ, USA).

### Cell culture

The human U87 and U251 glioblastoma cell lines (ATCC) were cultured in DMEM-F12, supplemented with B27 (Cat No. 17504-044; Gibco, Gaithersburg, MD, USA; 1:50) in the presence of 20 ng/ml epidermal growth factor (EGF; Cat No. E4127; Sigma Aldrich, St. Louis, MO, USA) and basic fibroblast growth factor (bFGF; Cat No. F0291; Sigma Aldrich) at 37 °C under 5% CO_2_ to form neurospheres. The medium was replaced every 2 days. After 1 week, cells were treated with 0.25% trypsin (Cat No. 25200-056; Gibco) in HBSS (Cat No. 14170-112; Gibco) for 5 min at 37 °C. Trypsin was washed out and the cells were mechanically dissociated and plated for distinct assays.

### PrP^C^/HOP silencing

U87 cells were infected with lentiviral particles carrying two constructions targeted to the human PrP^C^ sequence [15]. Efficiency was low for shRNA-PrP1 and high for shRNA-PrP2. Therefore, shRNA-PrP1 was used to silence PrP^C^ to intermediary levels, while shRNA-PrP2 was used for low PrP^C^ expression. Replication-deficient lentiviral particles were produced in HEK293FT cells using the ViraPower Lentiviral Expressing System (Invitrogen) according to the manufacturer’s instructions. Dr. Andrew Hill (La Trobe University, Australia) kindly provided the constructs for shRNA-PrP^C^ sequences. The following pLenti constructs were used for the shRNA-PrP^C^ sequence:

- shRNA-PrP1: 5′-caccgcgtcaatatcacaatcaagccgaagcttgattgtgatattgacgc-3′
- shRNA-PrP2: 5′- caccagaacaacttcgtgcacgactcgaaaagtcgtgcacgaagttgttc-3′

Stable silencing of HOP was performed using MISSION® shRNA (Sigma Aldrich) according to the manufacturer’s instructions using the following sequences: TRC 2.0; NM_006819 – Mission – SIGMA/TRCN0000243096 and TRCN0000243099.

### CRISPR/Cas9 for PrP^C^ knockout

The human PrP^C^ gene sequence (NM_000311.3) was used to design the guide RNA using the Optimized CRISPR Design (http://crispr.mit.edu/). gRNA sequences selected were: Hu PrP^C^ (Top1) CACCGgctgggggcagccgatacccg/Hu PrP^C^ (Bottom1) AAACcgggtatcggctgcccccagcC. The gRNAs were phosphorylated, annealed, and cloned into px330-U6-GFP vector (Addgene) according to the Addgene website instructions. The construct was sequenced, transfected into the U251 cell line with lipofectamine 2000 (Invitrogen) following the manufacturer’s instructions, and the clones were isolated by serial dilution.

### Flow cytometry analysis

Cells (10^6^) were dissociated, washed twice with phosphate-buffered saline (PBS), and incubated with anti-PrP^C^ [23], anti-CD133 (Cat No. 130-090-852; Miltenyi, Auburn, CA, USA), CD15 (Cat No. FCMAB182F; Millipore, Temecula, CA, USA), anti-E-cadherin (Cat No. 610181; BD Bioscience, San Diego, CA, USA), and anti-integrin α6 (Cat No. ab97760; Abcam, Cambridge, UK) antibodies, all at 1:50 dilution in 0.5% bovine serum albumin (BSA) in PBS for 60 min at 4 °C. After washing, samples were incubated with anti-mouse IgG Alexa-488/PE (Cat No. A21200; Invitrogen, Carlsbad, CA, USA; 1:200) or with anti-rabbit IgG Alexa-488/405 (Cat No. A21441; Invitrogen; 1:200) antibodies for 60 min at 4 °C. For PrP^C^ internalization assays, cells were pre-incubated for 40 min in 250 μM solution of CuSO_4_ in PBS plus 5% BSA at 37 °C, followed by antibody incubation. Only secondary staining was used for the negative control. Cells were analyzed by flow cytometry for forward scatter, side scatter, and fluorescence (FACSCanto II; BD Biosciences).

### Immunoblotting assays

For the analysis of neurosphere protein extracts, cells were cultured overnight (2 × 10^5^ cells/well), starved for 24 h, treated with serum or recombinant HOP for 15 min, and washed with cold PBS. Protein extracts were prepared in RIPA buffer with protease/phosphatase inhibitors, centrifuged (10,000 × *g*), and then loaded (5 μg) onto a 10% SDS-PAGE gel, followed by immunoblotting with polyclonal anti-HOP (1:10,000) [23], anti-phospho Erk1/2 (Cat No. 4370S; Cell Signaling, Danvers, MA, USA; 1:4000), anti-Erk1/2 (Cat No. 4695S; Cell Signaling; 1:4000) or anti-PrP^C^ [23]. Anti-GAPDH (Cat No. G9545; Sigma Aldrich) or anti-actin antibodies (Cat No. A2103; Sigma Aldrich) were used as protein loading controls.

### Immunofluorescence staining

For PrP^C^ and Oct4 staining, whole neurospheres were harvested, fixed in 4% paraformaldehyde, and paraffin-embedded. For Ki67 experiments, xenografts (Balb/c nude mice) were resected, fixed in 4% paraformaldehyde, and paraffin-embedded. Slides with 3-μm sections were prepared for immunofluorescence. Sections were incubated in xylol at 60 °C for paraffin removal and immersed in citrate buffer, pH 6.0, for 1 h at 96 °C for antigen retrieval. Sections were then blocked for 1 h at room temperature (RT) in 5% BSA in PBS and incubated overnight at RT with anti-PrP^C^ (1:50) [23] or anti-Oct4 (Cat No. 2840S; Cell signaling; 1:50) in 1% BSA in PBS. After washing, slides were incubated for 1 h at RT with anti-mouse Alexa-488 (Cat No. A21202; Invitrogen; 1:1000) or anti-rabbit Alexa-546 (Cat No. A10040; Invitrogen; 1:1000), and stained with TO-PRO (Cat No. T3605; Molecular Probes, Eugene, OR, USA) for nuclei. For other markers, whole neurospheres were harvested, plated on coverslips previously treated with poly-l-lysine and fixed in 4% paraformaldehyde. Coverslips were blocked for 1 h at RT with 5% BSA plus 0.3% triton in PBS. Coverslips were incubated overnight at RT with anti-PrP^C^ (1:100) and anti-HOP (1:100) [23], anti-nestin (Cat No. N5413; Sigma Aldrich; 1:100), anti-Musashi1 (Cat No. 5663P; Cell Signaling; 1:100), anti-Sox2 (Cat No. ab75485; Abcam; 1:100), anti-βIII tubulin (Cat No. 5568S; Cell Signaling; 1:100), anti-β catenin (Cat No. ab32572; Abcam; 1:100), anti-CD133 (Cat No. MAB4399; Millipore; 1:100), anti-GFAP (Cat No. Z0334; Dako, Cambridge, UK; 1:100) or anti-E-cadherin (Cat No. 610181; BD Bioscience; 1:100) in 1% BSA 0.1% triton in PBS. After washing, coverslips were incubated for 1 h at RT with anti-mouse Alexa-488 (Cat No. A21202; Invitrogen; 1:1000) or anti-rabbit Alexa-546 (Cat No. A10040; Invitrogen; 1:1000), and stained with TO-PRO (Cat No. T3605; Molecular Probes) or DAPI (Cat No. D1306; Invitrogen) for nuclei. Cells were imaged on a Leica TCS SP2 II laser scanning confocal system.

### Cell proliferation assay

Whole neurospheres were harvested and plated on coverslips previously treated with poly-l-lysine in DMEM-F12 supplemented with B27 at 37 °C. Control cells were treated only with the growth factors (20 ng/ml) EGF (Cat No. E4127; Sigma Aldrich) and bFGF (Cat No. F0291; Sigma Aldrich), and for HOP treatments cells were treated with growth factors and recombinant HOP and/or HOP peptides (1 μM) for 24 h. Cells received a 60-μM BrdU pulse 3 h prior to fixation in 4% paraformaldehyde. Fixed cells were treated with HCl 2 N for 30 min, washed with borate buffer, and permeabilized with 0.3% Triton X-100 in PBS for 15 min. Cells were blocked in 0.3% Triton 5% BSA in PBS for 1 h and stained with biotin-conjugated anti-BrdU (Cat No. MAB3262B; Millipore; 1:100), Strepta-AlexaFluor-546 (Cat No. S11225; Molecular Probes; 1:500), and DAPI (Cat No. D1306; Invitrogen; 1:500) for nuclei. Images were taken from at least four microscopic fields (Zeiss AxioVertA1) for each duplicate per treatment and analyzed on ImageJ software (NIH). The percentage of BrdU-positive nuclei in the total number of nuclei (DAPI) was calculated. HOP-silenced populations were plated 24 h prior to performing the transfection and proliferation assays (as described above). For colorimetric assays, cells were treated with growth factors and recombinant HOP and/or HOP peptides (1 μM) for 24 h and the Cell Proliferation ELISA BrdU kit (Cat. No. 11647229001; Roche, Indianapolis, IN, USA) was utilized according to the manufacturer’s instructions.

### Clonal density assay

Neurospheres were treated with 0.25% trypsin (Cat No. 25200-056; Gibco) in HBSS (Cat No. 14170-112; Gibco) for 20 min at 37 °C. Trypsin was washed out and cells were mechanically dissociated. In each well (96-well plate), 200 cells were plated in triplicates per condition (control or treatment with 500nM recombinant HOP and/or HOP peptides). Cells were treated every 48 h for 1 week. Images were acquired through light microscopy (Zeiss PrimoVert) and the number and size of neurospheres were evaluated and compared between conditions with the ZEN software (Zeiss). The optimal concentration of HOP for self-renewal or proliferation assays was chosen based on previous experiments of dose-response curve (data not shown) or according to previous data [6], respectively.

### Migration assay

Neurospheres were plated on coverslips previously treated with poly-l-lysine and laminin-1 (5 μg/ml) in DMEM-F12 supplemented with B27, and the growth factors EGF (Cat No. E4127; Sigma Aldrich) and bFGF (Cat No. F0291; Sigma Aldrich) and 2% fetal bovine serum (FBS; Lot No. 003/14; Vitrocell, Campinas, Spain), and cultured for 24 h at 37 °C. Images were acquired through light microscopy (ZeissPrimoVert) and the halo of migration was compared to neurosphere radius to evaluate cell migration using ZEN software (Zeiss). For cell scratch assays, neurospheres were dissociated and plated 0.2 × 10^6^ per well (six-well plate), the scratch was performed, and images were acquired at 0 h and 24 h after the scratch. Images of three experimental replicates were acquired using Zeiss PrimoVert microscope and the distance of each scratch closure after 24 h was measured by comparing with the images at time 0 h using ZEN software (Zeiss).

### In vivo tumor growth

Institutional guidelines for animal welfare were followed and the study approved by the Animal Ethics Committee of the Institute of Biomedical Sciences/University of Sao Paulo (book 03, page 15, protocol number 002 of 04/03/2014). Neurospheres (1 × 10^6^) as single cells were injected subcutaneously into the flank of female Balb/C nude mice (12 weeks old) in PBS. Tumor growth was measured every 2 days and euthanasia by CO_2_ saturation was performed on day 10 after tumor detection. Tumors were resected and fixed in paraformaldehyde 4%.

### Statistical analysis

One-way analysis of variance (ANOVA) followed by Tukey’s post-hoc test was used for multiple comparisons. A *p* value <0.05 was considered statistically significant. The non-parametric Student’s *t* test was also used in migration assays. Mean values represent at least three independent data sets; error bars represent standard errors of the mean (SEM).

## Results

### Characterization of neurosphere culture from GBM cell line

**Fig. 1:**
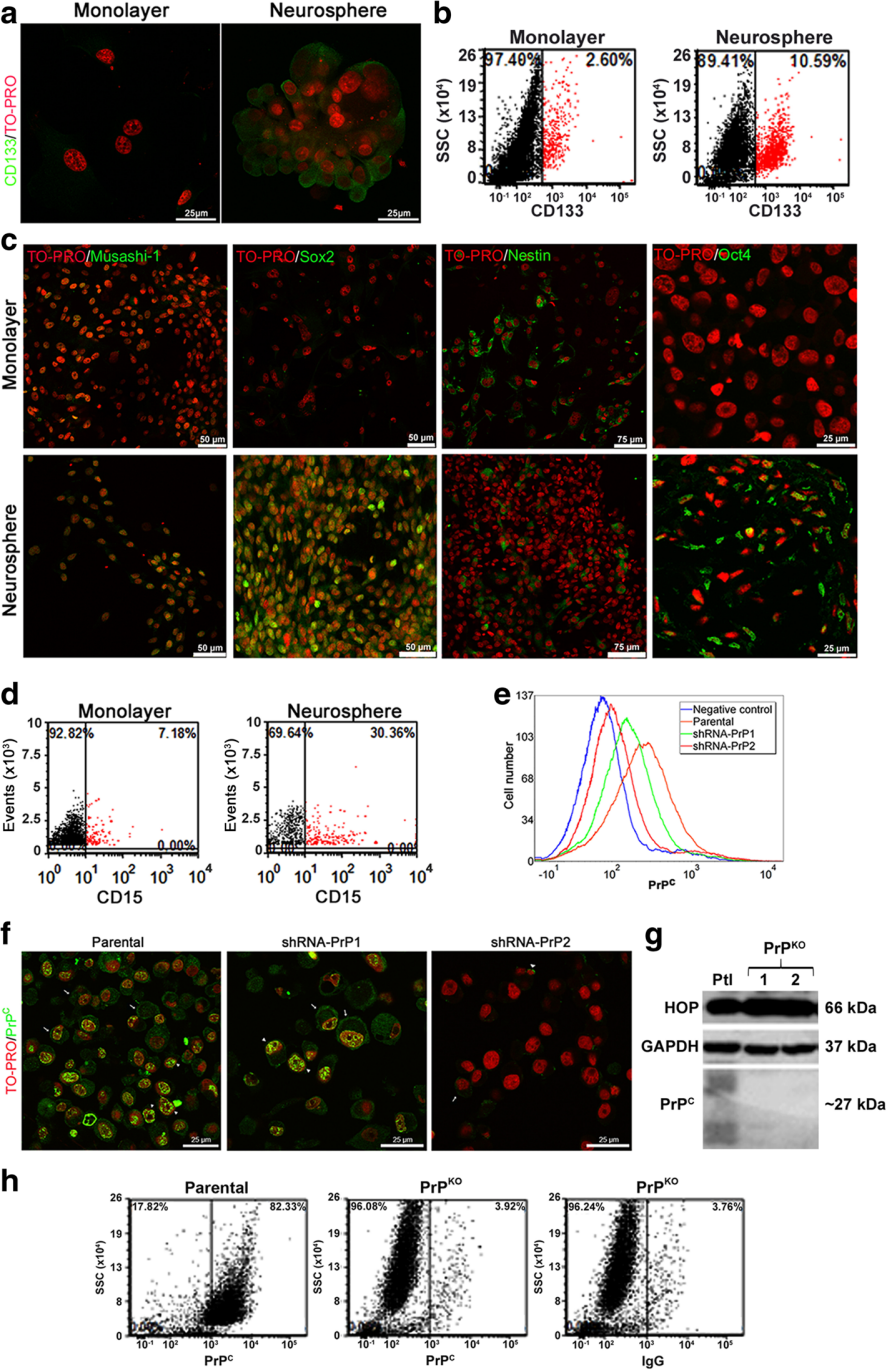
Characterization of glioblastoma U87 and U251 neurospheres. **a** Immunofluorescence for CD133 (*green*) in U87 cultured as monolayer plus serum (*left*) or neurospheres (*right*). Nuclei staining (TO-PRO) shown in *red*. **b** Dot plot for CD133 expression in monolayer cultured with serum (*left*) and neurospheres (*right*). CD133^+^ cells shown in *red* and CD133^–^ cells in *black* **. c** Immunostaining for the stem cells markers Oct4, Musashi-1, Sox2, and Nestin (*green*) in monolayer (*upper*) and neurospheres (*lower*), with nuclei staining (TO-PRO) shown in *red*. **d** Dot plot graph for CD15 expression in monolayer cultured with serum (*left*) and as neurospheres (*right*). CD15^+^ shown in *red* and CD15^–^ in *black*. **e** Cellular prion protein (*PrP* ^*C*^) expression assessed by flow cytometry in parental (*orange*), shRNA-PrP1 (*green*), or shRNA-PrP2 (*red*) populations. Negative control shown in *blue* (only secondary antibody staining). **f** Immunofluorescence for PrP^C^ (*green*), in parental (*left*), shRNA-PrP1 (*middle*), or shRNA-PrP2 (*right*) populations. *Arrow* indicates staining on the cell surface and *arrowhead* in the perinuclear region. Nuclei staining (TO-PRO) shown in *red*. **g** Immunoblot for Hsp70/90 organizing protein (*HOP*) (*top*) and PrP^C^ (*bottom*) expression in U251 knockout (*PrP* ^*KO*^) clones (1 and 2) compared to the parental (*Ptl*) population. GAPDH was used as the loading control. Note that the smear for PrP^C^ immunostaining is due to the different glycosylated isoforms. **h** Flow cytometry for PrP^C^ expression in the U251 populations parental (*left*) and U251 PrP-knockout clone 2 (PrP^KO^) (*middle*). IgG isotype (*right*) was used as the negative control

We compared the expression of several stem cell markers in monolayer and neurosphere cultures of the U87 glioblastoma cell line. Neurospheres showed higher expression of the stem cells markers CD15, CD133, Oct4, Musashi-1, and Sox2, suggesting enrichment in the number of stem-like cells and thus supporting the use of the neurosphere-formation assay as a model to study GSCs (Fig. 1a–d). However, U87 monolayer cultures and neurospheres presented similar expression of the neural precursor marker nestin (Fig. 1c).

### Effect of PrP^C^ modulation on the expression of stem-cell markers

Due to their roles in GBM and NSCs, PrP^C^ and HOP were proposed to modulate essential functions in GSC biology. To better understand these functions, an shRNA lentivirus system was used in the U87 cell line to silence PrP^C^ to intermediary (shRNA-PrP1) and low (shRNA-PrP2) levels (Fig. 1e and f). The CRISPR/Cas9 technique was used to generate PrP^C^ knockout U251 cells (Fig. 1g). U251 clone 2 presented clear depletion of PrP^C^ expression (PrP^KO^) and was selected to perform functional experiments (Fig. 1g and h). Both monolayer populations (shRNA-PrP1and shRNA-PrP2), previously used in [15], and also PrP^KO^ were cultured as neurospheres.

**Fig. 2:**
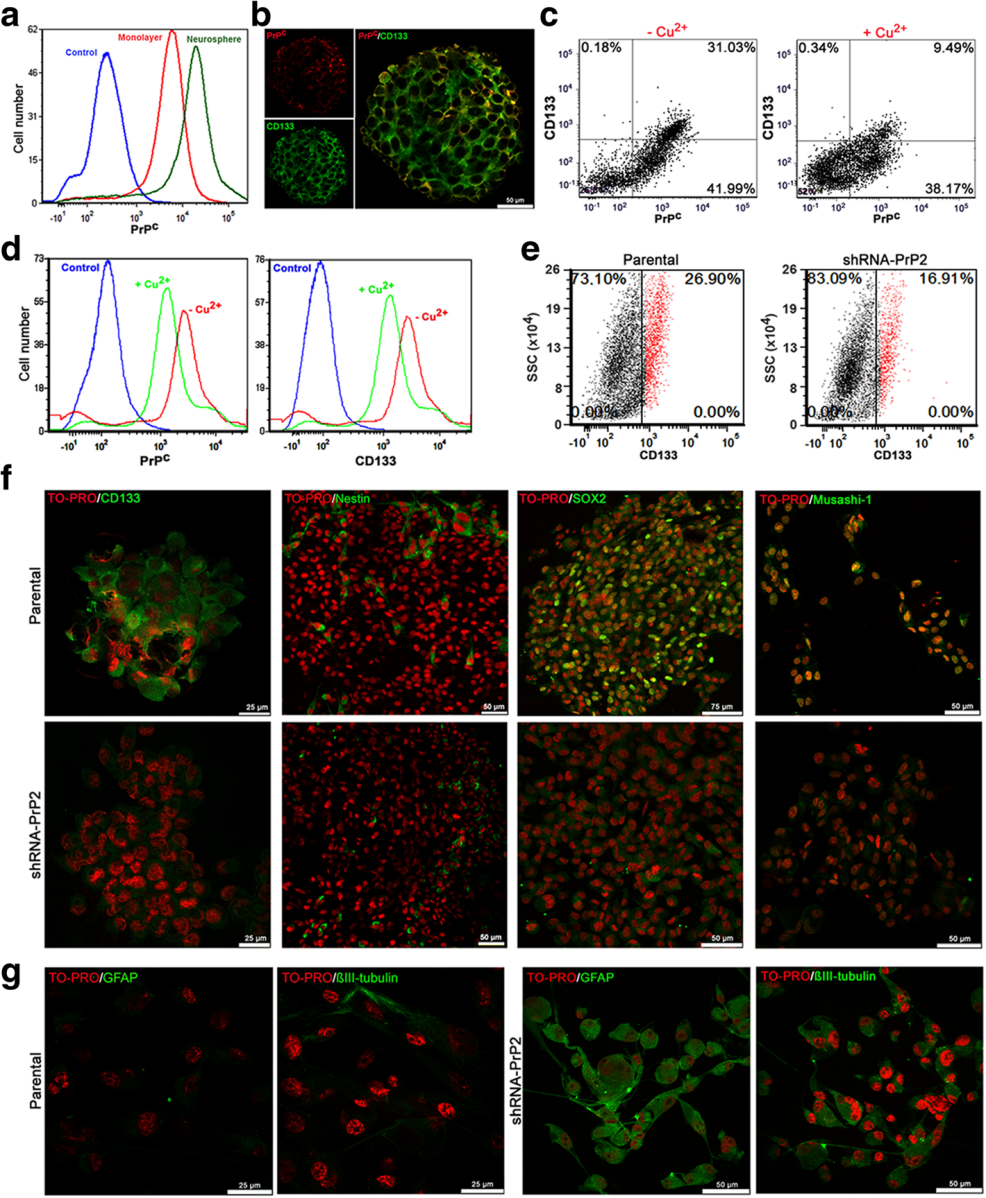
Stem cells marker expression in cellular prion protein (*PrP* ^*C*^)-depleted neurospheres. **a** PrP^C^ expression assessed by flow cytometry in parental monolayer (*red*) and neurosphere (*green*) cultures. Negative control shown in *blue* (only secondary antibody staining). **b** Immunofluorescence for PrP^C^ (*red*) and CD133 (*green*) in parental neurospheres shows co-localization on the cell surface. **c** Dot plot of CD133 and PrP^C^ expression in parental neurospheres in the absence (–Cu^2+^) or presence (+Cu^2+^) of CuSO_4_ 250 μM. **d** Histogram for PrP^C^ and CD133 in the absence (–Cu^2+^) and presence (+Cu^2+^) of CuSO_4_ 250 μM. Negative control shown in *blue* (only secondary antibody staining). **e** Dot plot of CD133 expression in parental (*left*) and shRNA-PrP2 (*right*) neurospheres. CD133^+^ shown in *red* and CD133^–^ shown in *black*. **f** Immunofluorescence for the stem cells markers musashi-1, nestin, Sox2, and CD133 (*green*) in parental (*upper*) and shRNA-PrP2 (*lower*) neurospheres. Nuclei staining (TO-PRO) shown in *red*. **g** Immunofluorescence for the cell differentiation markers GFAP and βIII-tubulin (*green*) in parental (*left*) and shRNA-PrP2 (*right*) neurospheres after 5 days of serum treatment. Nuclei staining (TO-PRO) shown in *red*

Compared to monolayer cells, PrP^C^ is upregulated in neurospheres (Fig. 2a); therefore, the detection of its expression may indicate tumor enrichment in stem-like populations. Additionally, the stem cells marker CD133 was co-expressed with PrP^C^ and co-localized partially on the plasma membrane of neurospheres (Fig. 2b), being internalized with PrP^C^ in the presence of copper presenting a similar endocytosis profile (Fig. 2c and d), suggesting that these molecules can form a functional complex on the membrane domain.

The stem cell marker CD133 expression is decreased in the U87 PrP2 population compared to the parental population (Fig. 2e), and Sox2 had higher expression in the parental population than in the PrP2 population (Fig. 2f). Musashi-1, on the other hand, presented different cellular locations: nuclei for parental population and cytoplasm for PrP2 (Fig. 2f). No significant difference in expression of the neural precursor marker nestin was observed between populations (Fig. 2f). Cellular differentiation markers, such as GFAP and βIII-tubulin, were highly expressed in PrP2 cells compared to parental neurospheres after serum stimulation (Fig. 2g). These findings reveal a potential role for PrP^C^ in the regulation of the GSC multipotent status.

### The PrP^C^-HOP complex modulates GSC proliferation

**Fig. 3:**
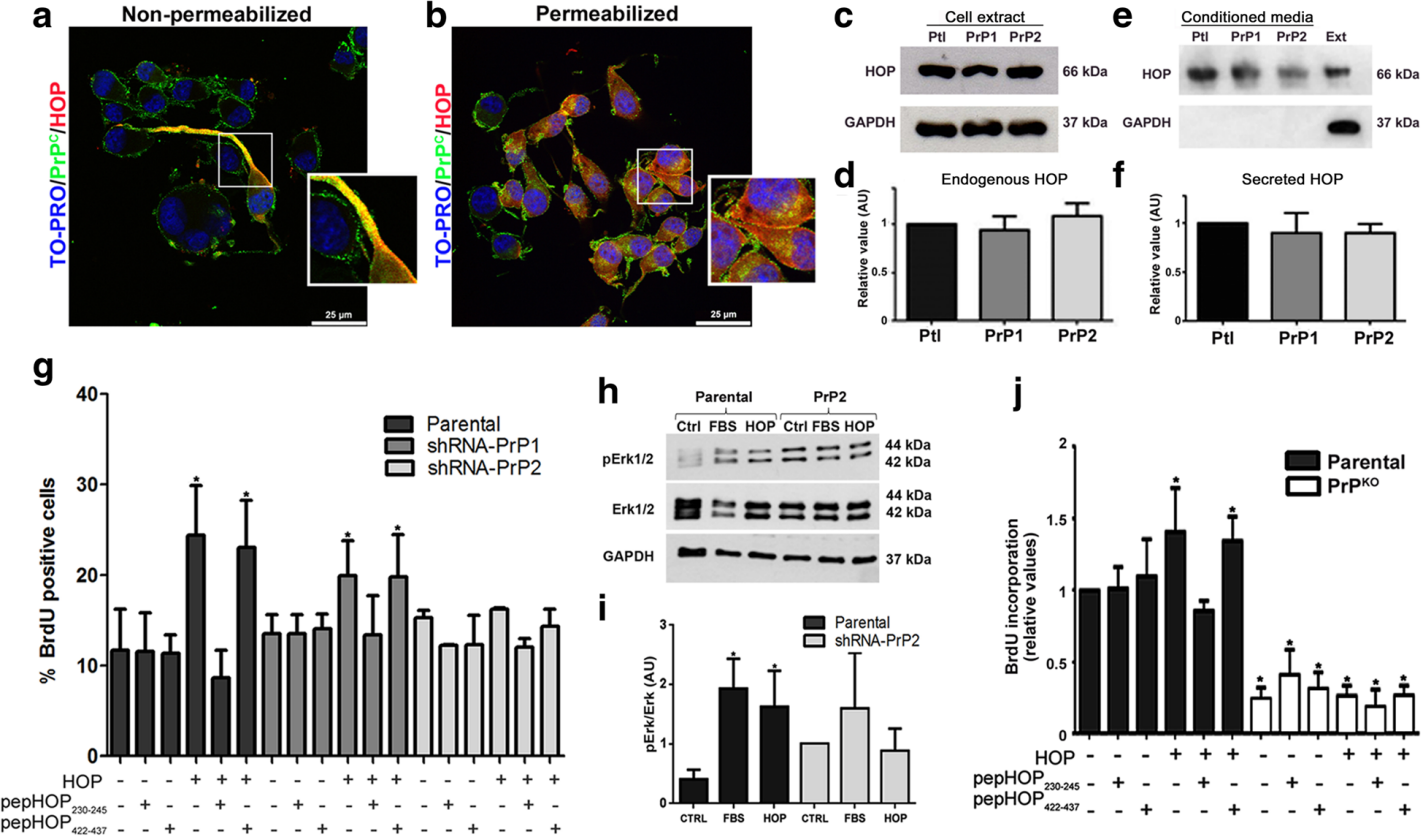
Hsp70/90 organizing protein (*HOP*) promotes proliferation of neurospheres dependent on cellular prion protein (*PrP* ^*C*^) by activating the Erk1/2 signaling pathway. Immunofluorescence for PrP^C^ (*green*) and HOP (*red*) in parental neurospheres shows co-localization on the cell surface in non-permeabilized cells (**a**) and expression of both proteins in the cytoplasm in permeabilized cells (**b**). Nuclei staining (TO-PRO) in shown in *blue*. A higher magnification field is shown in the inset. **c** Immunoblot for HOP in parental (*Ptl*), shRNA-PrP1 (*PrP1*), and shRNA-PrP2 (*PrP2*) neurospheres. GAPDH was used as the loading control. **d** Densitometry analysis of immunoblot; values of three independent experiments are expressed relative to control (parental). **e** Immunoblot of conditioned medium of parental (*Ptl*), shRNA-PrP1 (*PrP1*), and shRNA-PrP2 (*PrP2*) PrP^C^-depleted populations. Total protein extract (*Ext*). GAPDH immunodetection was used as cell lysis control. **f** Densitometry analysis of immunoblot; values of three independent experiments are expressed relative to control (parental). **g** U87 neurospheres treated with HOP and/or peptides pepHOP_230–245_ or pepHOP_422–437_ (1 μM), combined or alone, compared to untreated control. Percentage of BrdU-positive cells in all conditions in relation to total number of cells (*n* = 6, *p < 0.005). **h** Immunoblot of phosphorylated Erk1/2 and total Erk1/2 in parental and shRNA-PrP2 neurospheres for basal levels (*Ctrl*), fetal bovine serum (*FBS*), or recombinant HOP treatments (*HOP*) for 20 min. GAPDH was used as the loading control. **i** Densitometry of the relative values of ERK1/2 activation are represented by the ratio of p-ERK and total ERK1/2 in parental and shRNA-PrP2 neurospheres after treatment with recombinant HOP or FBS. **j** U251 neurospheres of parental and U251 knockout (*PrP* ^*KO*^) populations treated with HOP and/or peptides pepHOP_230–245_ or pepHOP_422–437_ (1 μM), combined or alone, compared to untreated control. BrdU-positive cells detected by spectrophotometry (*n* = 4, *p* < 0.05, ANOVA followed by Tukey post-hoc test)

Recently, our group described that the expression of PrP^C^ and HOP associates with GBM malignancy [15] and, considering that GBM contains stem cells implicated in tumor development, we evaluated the role of the PrP^C^-HOP interaction in GSC biology. We observed a clear co-localization of PrP^C^ and HOP on the cell surface of neurospheres (Fig. 3a) and both proteins are highly expressed in neurospheres (Fig. 3b). In addition, GSC neurospheres from both parental and shRNA-PrP^C^ populations present equivalent endogenous (Fig. 3c and d) and secreted (Fig. 3e and f) HOP levels, indicating that the expression pattern of HOP is not affected by PrP^C^ ablation in GSCs.

We evaluated the effect of exogenous (recombinant) HOP and synthetic peptides (pepHOP_230–245_ which mimics the PrP^C^ binding site, and peptide pepHOP_422–437_ used as the control) on the proliferation of parental, PrP1, and PrP2 neurospheres. After 24 h of treatment, there was a significant increase in proliferation of parental and of PrP1 neurospheres treated with HOP, compared to PrP^C^ silenced cells (PrP2) (Fig. 3g). On the other hand, populations pre-treated with pepHOP_230–245_ were unable to proliferate in the presence of HOP (Fig. 3g), suggesting that pepHOP_230–245_ is able to block the PrP^C^-HOP interaction and impair GSC proliferation. Moreover, we observed that HOP activates the Erk1/2 pathway only in neurosphere cultures expressing high levels of PrP^C^ (Fig. 3h and i). PrP^C^-depleted cells have increased basal levels of phospho-Erk1/2 (Fig. 3h and i), corroborating our previous data in primary cultures from PrP^C^ knockout mice and literature data in different cell types [24–26]. Similar results were obtained using an additional glioblastoma cell line (U251). As shown in Fig. 3j the positive HOP effect upon proliferation was also abrogated by HOP_230–245_ peptide in U251 PrP^C^-expressing cells, as well as being observed with U87 (Fig. 3g). Neurospheres from the PrP^C^ knockout U251 cell line present a very low proliferation rate when compared to parental cells (Fig. 3j). Together these findings indicate that PrP^C^-HOP interaction is able to sustain the proliferation of GSCs of distinct glioblastoma cell lines.

### HOP silencing impairs GSC proliferation

**Fig. 4:**
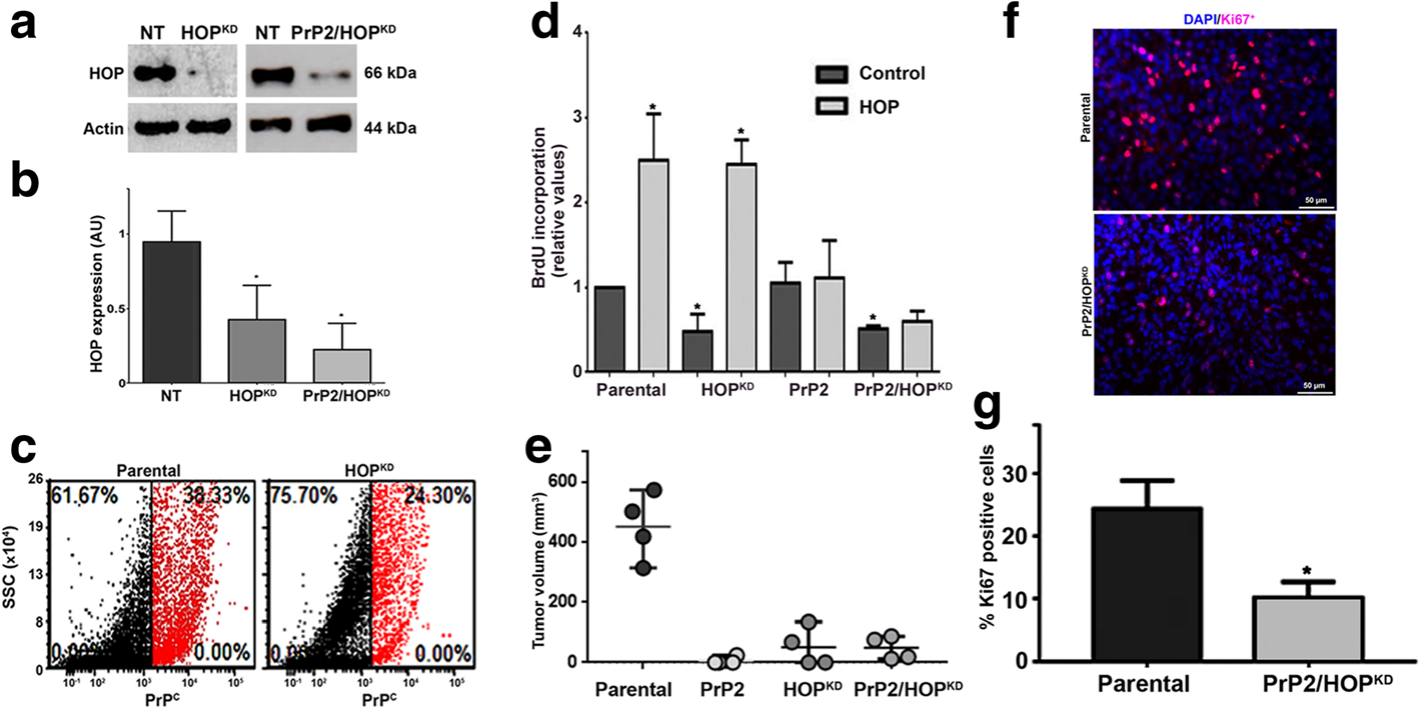
Cellular prion protein (*PrP* ^*C*^) and/or Hsp70/90 organizing protein (*HOP*) knockdown suppresses cell proliferation and tumor growth in vivo. **a** Immunoblot for HOP expression in U87 non-target (*NT*), shRNA-HOP (*HOP* ^*KD*^), or shRNA-PrP2/HOP (*PrP2/HOP* ^*KD*^) populations. Actin was used as the protein loading control. **b** Immunoblot densitometry analysis; values of three independent experiments are expressed relative to control (parental). **c** Dot plot for PrP^C^ expression of U87 parental and HOP^KD^ populations. PrP^C+^ cells shown in *red* and PrP^C–^ cells shown in *black*. **d**. Colorimetric BrdU incorporation assay in U87 parental, HOP^KD^, PrP2, or PrP2/HOP^KD^ populations. Values of four independent experiments are expressed relative to control (parental). *p < 0.05, ANOVA followed by Tukey post-hoc test. **e** U87 neurospheres cells from parental, PrP2, HOP^KD^, or PrP2/HOP^KD^ populations (1 × 10^6^ cells) were implanted into the flank of nude mice and the tumor growth was monitored daily. Data represent tumor volume on day 10 after tumor detection (*n* = 4, *p < 0.05, ANOVA followed by Tukey post-hoc test). **f** Tumors were resected, fixed, paraffin embedded, and immunostained for Ki67. Representative images of Ki67 labeling (*red*) and DAPI (nuclei, *blue*). **g** Values represent the percentage of Ki67-positive cells relative to total number of cells (nuclei, DAPI staining)

To determine the contribution of HOP towards GSC proliferation, we silenced HOP expression in parental and PrP2 populations. Decreased expression of HOP was confirmed by immunoblotting (Fig. 4a and b). The expression of PrP^C^ was also addressed in HOP knockdown cells (HOP^KD^) and showed a slight decrease compared to the parental population (Fig. 4c). The proliferative basal levels were affected in both HOP^KD^ and PrP2/HOP^KD^ populations (Fig. 4d); however, the treatment with recombinant HOP is able to rescue the proliferation phenotype in PrP^C^-positive cells (Fig. 4d). Together, these results indicate both PrP^C^ and HOP as key players in the regulation of GSC proliferation.

### PrP^C^ and HOP depletion decreases GSC tumorigenicity in vivo

Neurosphere cells expressing different levels of PrP^C^ and/or HOP were injected subcutaneously into Balb/c nude mice flanks and tumor growth was evaluated. As depicted in Fig. 4e, cells expressing PrP^C^ grew rapidly, while ablated cells for PrP^C^ (PrP2) and/or HOP (HOP^KD^) showed slower or no tumor formation. Histological sections of resected tumors showed that PrP^C^ or HOP silencing caused cell proliferation reduction, as depicted by Ki-67 nuclear immunostaining (Fig. 4f and g). These data support our in vitro assays and highlight the importance of PrP^C^ and HOP in tumor maintenance through the modulation of GSC proliferation.

### Self-renewal of GSCs depends on PrP^C^ expression and its interaction with HOP

**Fig. 5:**
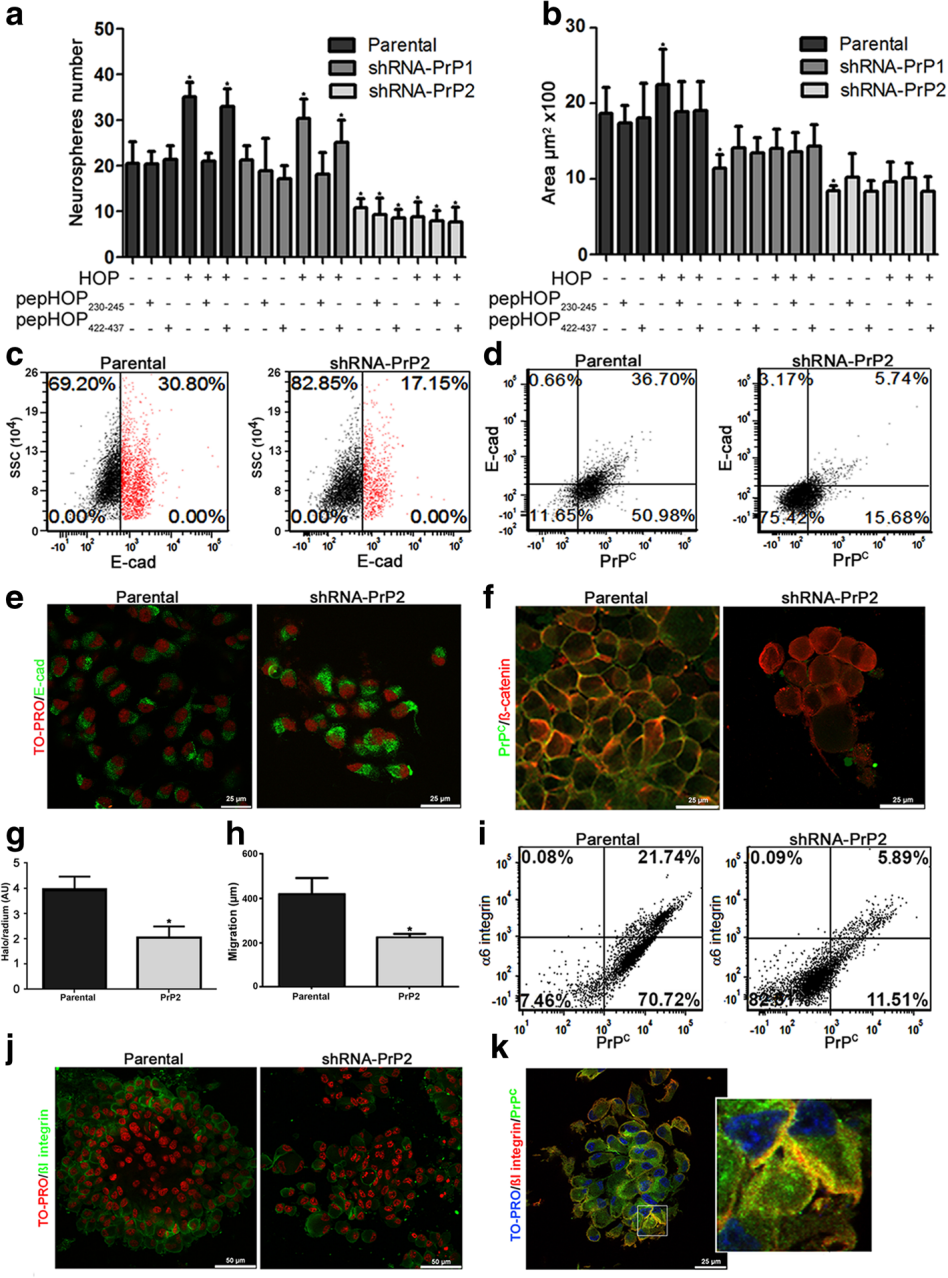
Cellular prion protein (*PrP* ^*C*^) promotes GSC self-renewal binding HOP and modulates cell surface adhesion molecule stability. Neurosphere number (**a**) or size (**b**) after 1 week treatment every 48 h with Hsp70/90 organizing protein (*HOP*) and peptides pepHOP_230–245_ and pepHOP_422–437_ (1 μM), combined or alone (500nM), compared to control (*n* = 6, *p < 0.05, ANOVA followed by Tukey post-hoc test). **c** Dot plot of E-cadherin expression in parental and shRNA-PrP2 neurospheres. E-cad^+^ cells shown in *red* and E-cad^–^ cells shown in *black*. **d** Dot plot of E-cadherin and PrP^C^ expression in parental and shRNA-PrP2 neurospheres. **e** Immunofluorescence for E-cadherin (*green*) in parental and shRNA-PrP2 neurospheres, showing expression on the cell surface (parental) and in the perinuclear region (shRNA-PrP2). Nuclei (TO-Pro) stain shown in *red*. **f** PrP^C^ (*green*) and β-catenin (*red*) expression and co-localization (*yellow*) of parental and shRNA-PrP2 neurospheres. **g** Migration assay, ratio between cell migration distance (halo), and neurosphere size for parental and shRNA-PrP2 neurospheres 24 h after plating on laminin-1 (*n* = 3, *p < 0.05). **h** Cell scratch assay; images of three experimental replicates were acquired and the distance of each scratch closure after 24 h was measured by comparing with the images at time 0 h for parental and shRNA-PrP2 neurospheres plated on laminin-1 (*n* = 4, *p < 0.05). **i** Dot plot of α6 integrin and PrP^C^ expression in parental and shRNA-PrP2 neurospheres. **j** Immunofluorescence for β1 integrin (*green*) of parental and shRNA-PrP2 neurospheres. Nuclei (TO-PRO) stain shown in *red*. **k**. PrP^C^ (*green*) and β1 integrin (*red*) expression and co-localization (*yellow*) of parental and shRNA-PrP2 neurospheres. Nuclei (TO-PRO) stain shown in *blue*; a higher magnification is shown in the inset

Since the proposal that NSCs originate GSCs, important data describing the induction of NSC self-renewal by HOP-PrP^C^ interaction has emerge, supporting a function for PrP^C^ and HOP in GSC biology [6]. Thus, self-renewal was evaluated by formation of secondary neurospheres in clonal density assays. Cells expressing lower PrP^C^ levels (PrP2 population) formed fewer colonies when compared to parental and PrP1 populations (Fig. 5a). HOP increased the number of neurospheres in parental and PrP1 populations when compared to untreated cells. However, any HOP effect was observed in the PrP2 population that lacked PrP^C^ (Fig. 5a). These results indicate that PrP^C^ expression is required for self-renewal and is also necessary to mediate HOP activity. Neurosphere formation mediated by HOP-PrP^C^ interaction was abrogated in the presence of pepHOP_230–245_, which mimics and competes for the PrP^C^ binding site. The control peptide pepHOP_422–437_ (Fig. 5a) did not have any effect.

As previously described, PrP^C^ ablation decreases neurosphere number (Fig. 5a) probably by downregulating essential molecules involved in stem cell maintenance (Fig. 2), supporting its previously suggested role in stemness. The smaller size of neurospheres formed by PrP^C^-depleted cells (compared to their counterparts) suggests a putative role for PrP^C^ in GSC cell adhesion maintenance (Fig. 5b; Additional file 1: Figure S1A). To address this issue, the expression of E-cadherin and β-catenin, key components of adherens junctions, was evaluated in PrP2 neurospheres and parental control populations. The expression of E-cadherin is associated with PrP^C^ on the cell surface; this was more obvious for the parental population, where there was higher expression of E-cadherin (Fig. 5c) and a clear double staining for both proteins in neurosphere cells (Fig. 5d). Although the cell membrane of parental cells had high E-cadherin expression, PrP2 had abundant cytoplasmic E-cadherin (Fig. 5e). Parental and PrP2 neurospheres expressed β-catenin abundantly at similar levels. However, there was an intense co-localization of β-catenin and PrP^C^ in the same membrane domain of parental neurospheres, while in PrP2 neurospheres the β-catenin expression was diffuse (Fig. 5f). This may suggests that PrP^C^ plays a role in E-cadherin recruitment to the cell membrane and, consequently, on β-catenin engagement.

Due to its putative function as a scaffold protein and multiprotein assembly platform on the cell surface, we also tested if additional anchorage-dependent cell processes, such as migration, was altered in PrP^C^-depleted cells. Additionally, migration on laminin is impaired in PrP^C^-silenced cells compared to parental neurospheres (Fig. 5g and h; Additional file 1: Figure S1B). Moreover, the surface expression of integrin α6β1, a laminin receptor, was verified. Integrin α6 expression is clearly more evident in parental neurospheres compared to PrP2 (Fig. 5i) and is co-expressed with PrP^C^, while integrin β1 expression is apparently similar in both populations (Fig. 5j). However, a high co-localization of integrin β1 and PrP^C^ was observed (Fig. 5k), suggesting that PrP^C^ plays a key function in enhancing cell surface stability of cell adhesion molecules, thereby modulating the invasive process.

## Discussion

In this study, the role of the PrP^C^-HOP complex in the regulation of GSC biology was evaluated. First, we demonstrated that our model of neurospheres expressed several markers of stemness. The expression of CD15, CD133, Oct4, Musashi-1, and Sox2 was increased in neurospheres compared to a non-stem condition (monolayer), validating our experimental model (Fig. 1). PrP^C^ expression was higher in neurospheres than in monolayer cultures (Fig. 2a) and, since the enrichment of GBM cultures with stem cells leads to more malignant tumors in vivo [27], these data support previous results from our group which show that PrP^C^ expression is correlated with tumor aggressiveness [6, 15].

PrP^C^ expression seems to be associated with stem-like properties, since its silencing led to a differentiated expression of stem cell markers. Compared to parental neurospheres, CD133 expression decreased and Sox2 expression became undetectable in PrP^C^-depleted populations (Fig. 2e and f). Previous studies have shown that CD133 and Sox2 are exclusively expressed at perinecrotic and perivascular regions associated with stem-like cell pools, and that nestin and Musashi-1 are homogeneously expressed across the tumor, identifying precursors [28]. PrP2 neurospheres expressed Musashi-1 in the cytoplasm in contrast to parental neurospheres, where it segregated preferentially to cell nuclei (Fig. 2f). Nuclear expression of Musashi-1 has been associated with activation of the Notch pathway in gliomas [29, 30] which, in turn, may lead to increased tumor malignancy via induction of proliferation and therapy resistance [31]. The expression of nestin was similar in parental and PrP2 neurospheres (Fig. 2f). Expression of differentiation markers is more evident in PrP2 neurospheres compared to the parental population after serum stimuli (Fig. 2g). Together, our results suggest that parental neurospheres have a stem-like phenotype with some precursor cells, and that the PrP2 population has precursors and cells committed to a specific phenotype. Indeed, the function of PrP^C^ in stem cell biology has been broadly studied. PrP^C^ has been described as an important molecule for neural commitment and for the proliferation of precursors [32]. In tumor stem-like cells, PrP^C^ promotes proliferation and migration [11]. PrP^C^ interacts with the cell surface protein CD44, a marker for several types of cancer stem-like cells [33] that associates with tumor-initiating and metastatic capacities and promotes epithelial-mesenchymal transition (EMT) and tumor growth after resection [34].

A possible interaction between PrP^C^ and CD133 was also observed given their co-expression and localization on the cell surface of parental neurospheres (Fig. 2b and c). PrP^C^ and CD133 were previously shown to localize to the same membrane domains (lipid rafts), modulating differentiation and stemness, respectively [35]. Additionally, a reduction in cell surface expression of CD133 associated with PrP^C^ was observed after copper stimulus, suggesting PrP^C^ as a carrier for CD133 internalization (Fig. 2c and d). PrP^C^ is constitutively endocytosed via clatrin-coated pits [36] and copper ions reversibly stimulate this endocytic pathway [37]. CD133 has been shown to affect the clathrin-endocytosis process [38] and trafficking down the endosomal and lysosomal pathway for degradation [39]. Remarkably, the cytoplasmatic domain of CD133 binds β-catenin and the downregulation of CD133 increases β-catenin degradation and impairs tumor growth in vitro and in vivo [39]. Indeed, the β-catenin localization was perturbed in PrP^C^-depleted cells (Fig. 5f), suggesting the central role of PrP^C^ in the stabilization of the signaling module on the cell surface.

PrP^C^-depleted populations formed less secondary neurospheres (Fig. 5a), suggesting that withdrawing PrP^C^ impairs self-renewal ability. This supports the hypothesis that PrP^C^ can act as an important player in stemness maintenance and that its dowregulation induces cell line commitment, corroborating recent evidence that showed a less oncogenic phenotype in PrP^C^-depleted GSCs [40]. Thus, it is possible that PrP^C^ acts as an essential molecule for GSC biology and is capable of maintaining an undifferentiated state in this GBM subpopulation and, since its expression may indicate tumor enrichment with stem-like cells, it may be used as a tumor progression marker. Interesting, the effect of temozolomide, a common chemotherapeutic agent for brain tumors, is enhanced in PrP^C^-depleted glioma cells, supporting PrP^C^ as an effective target for GBM [41].

As a number of studies show that PrP^C^ acts as a scaffold protein, assembling signaling platforms on the plasma membrane to elicit several biological processes including in stem cells (reviewed in [8, 42]), we looked for alterations in the expression of cell adhesion proteins on the cell surface of GSCs. We observed a decrease in E-cadherin and integrin α6 expression on the cell surface of PrP^C^-silenced populations (Fig. 5c, d and i), and detected E-cadherin in the cytoplasm (Fig. 5e). The expression of these proteins was also associated with PrP^C^, as E-cadherin- or integrin α6-positive cells were almost exclusively positive for PrP^C^ (Fig. 5d and i). Integrin β1 expression was detected in both parental and PrP2 neurospheres, and co-localized with PrP^C^ on the cell surface (Fig. 5j and k). In addition, GSC migration on laminin was impaired in PrP^C^-depleted cells (Fig. 5g and h). Indeed, it has been demonstrated that PrP^C^ participates in E-cadherin recruitment to the cell surface [43] with no significant differences verified in transcripts levels [43, 44]. Interestingly, it was demonstrated that PrP^C^-null mice present increased paracellular permeability, with lower levels of E-cadherin, desmoplakin, occluding, and other proteins related to cell-cell junctions in intestinal tissues [45]. Furthermore, it was reported that PrP^C^ is able to regulate β1 integrin adhesiveness modulating ligand-induced changes in integrin activation [46]; however, its depletion had no effect on total β1 integrin expression levels [47]. These data suggest that PrP^C^ may be capable of recruiting cell adhesion molecules to the cell surface of GSCs, raising the hypothesis of PrP^C^ modulating invasion-related processes.

We also reported that GSCs have high expression of HOP and PrP^C^ (Fig. 3b) and described the HOP-PrP^C^ engagement on their plasma membrane (Fig. 3a). HOP expression and secretion was similar in parental, PrP1, and PrP2 populations (Fig. 3c–f), as demonstrated previously by Santos and collaborators in murine wild-type and PrP^C^-null neurospheres [6]. We demonstrated that HOP is able to modulate GSC proliferation (Fig. 4d) and self-renewal, depending on its interaction with PrP^C^ (Figs. 3g, 4d, and 5a); inhibiting the PrP^C^-HOP interaction with a HOP peptide, which mimics the PrP^C^ binding site, abrogates the effects of recombinant HOP (Figs. 3g and 5a). Similar effects were also observed in another glioblastoma cell line (U251) confirming the importance of these complexes in GSC biology (Fig. 3j). Another HOP peptide (anti-TPR), which inhibits the HOP-Hsp90 interaction, has been described to induce cell death in several cancer cell lines [48] and produce a cytotoxic effect in glioblastomas [49], highlighting HOP as a potential target for GBM therapy. Previous reports [15] as well as data from this study (Fig. 3e and h) also described that PrP^C^ and secreted HOP interact on the cell surface of human glioblastoma cell lines and modulate GBM progression by promoting proliferation through activation of the Erk1/2 pathway [16]. Remarkably, a higher basal phosphorylation of Erk was found in PrP^C^-depleted cells when compared to their counterparts (Fig. 3h and i), confirming our prior data in retinae [24] and hippocampal neurons [25], as well as those from other authors in adult brain and cerebellum extracts [26]. This feature suggests that high activity of Erk could be a compensatory effect for prion protein ablation [42]. Indeed, the targeting of stress response proteins has remarkable potential for the development of molecular treatments. Simultaneous inhibition of expression of both HOP ligands Hsp70 and Hsp90 reduced proliferation and promoted apoptosis in GBM cell lines in vitro [50]. Furthermore, HOP performs an important function in compensatory mechanisms of stress responses in tumor cells [51], further supporting its relevance in GBM maintenance.

Finally, we observed that PrP^C^ and/or HOP knockdown affects the proliferative and tumorigenic capacity of GSCs (Fig. 4e and f) in vivo, and supports PrP^C^ and HOP molecules as potential new targets for developing more efficient therapeutic strategies.

## Conclusions

**Fig. 6:**
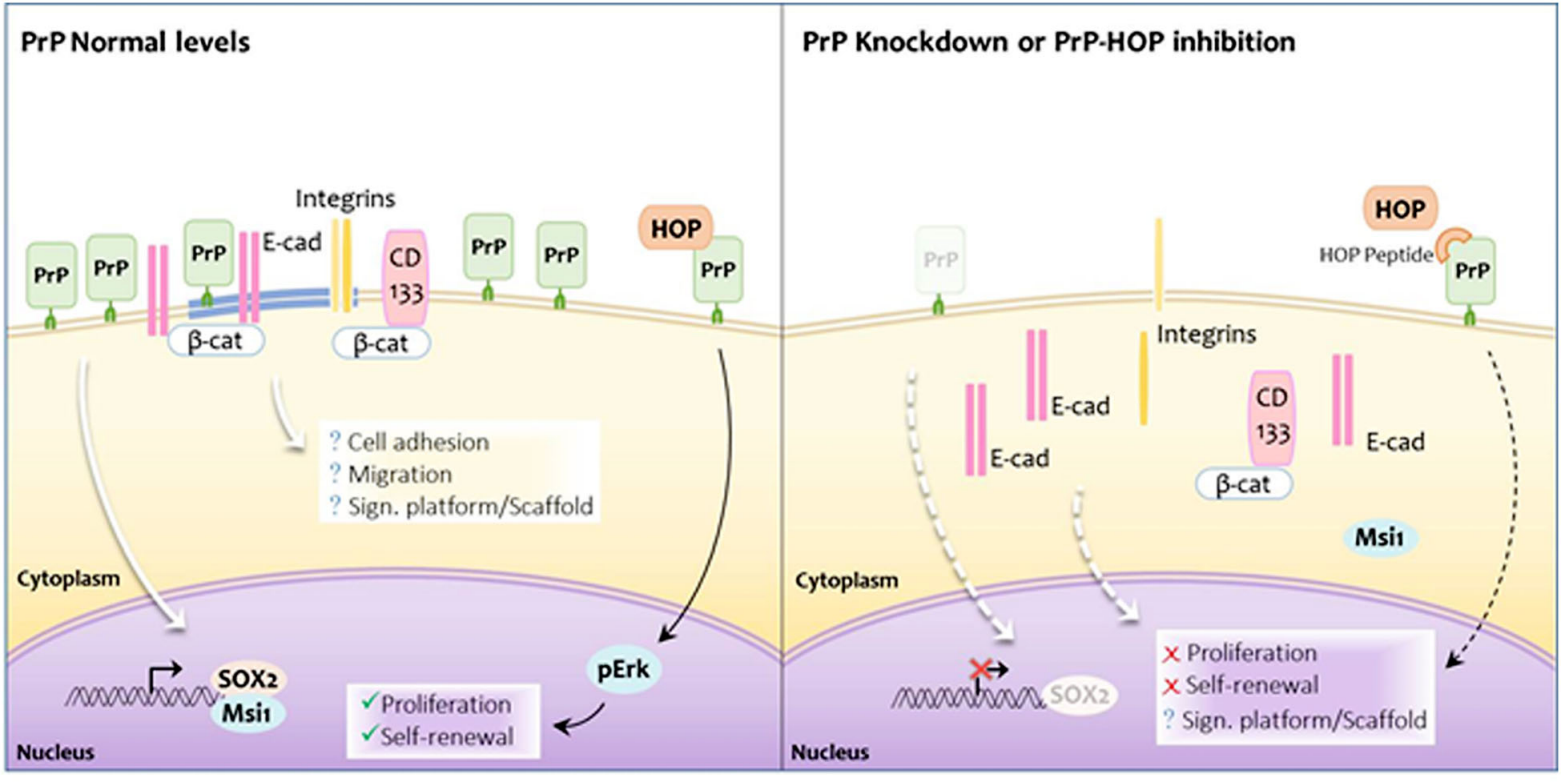
Model of prion protein (*PrP*) as a scaffold protein modulating GSC biology. Scheme illustrating how PrP^C^ may act as a scaffold protein, regulating stemness (dowregulation of CD133 and Sox2 and Msi1 sub-localization altered), recruiting cell adhesion molecules (E-cadherin and integrin α6β1), binding CD133-β-catenin to the plasma membrane, and modulating GSC proliferation and self-renewal through its interaction with Hsp70/90 organizing protein (*HOP*). Blockage of PrP^C^-HOP interaction with HOP peptide impairs the binding of HOP and PrP^C^ and, consequently, Erk1/2 activation, affecting proliferation

Here, we reported that HOP promotes GSC proliferation and self-renewal by interacting with PrP^C^ and that silencing PrP^C^ significantly impairs GSC self-renewal. PrP^C^-silenced cells have lower expression of stem cell markers and increased differentiation, suggesting that PrP^C^ plays a role in maintenance of an undifferentiated state in GSCs. HOP and PrP^C^ ablation suppresses the malignancy of GBM cells in vivo and has potential application as a therapeutic target in glioblastoma. In addition, PrP^C^ could play a role in the expression and cell surface localization of cell adhesion proteins, participating in cell migration mechanisms and, consequently, in invasion. Our results suggest that one mechanism by which PrP^C^ governs GSC biology is through its role as a cell surface scaffold protein assembling a dynamic signaling platform and also interacting with soluble partners, such as HOP, to maintain stemness status (Fig. 6).

Major commitments for developing novel therapeutic strategies for GBM are under way, as this is an extremely aggressive type of cancer. In particular, several attempts to therapeutically eradicate GSCs have been made, as these cells have well-established characteristics in tumors. Our findings support the concept that PrP^C^, HOP, and their complex are important for GSC biology, regulating essential mechanisms for tumor maintenance. Therefore, they represent a novel target for developing new treatments for GBM or for improving the efficacy of current therapies by targeting GSCs.

## Additional file

Additional file 1: Figure S1. Representative images of self-renewal and migration assays. (A) Representative image of self-renewal assay. Left panel: neurosphere growth in parental, shRNA-PrP1 (PrP1), and shRNA-PrP2 (PrP2) populations. Right panel: neurosphere size for the parental population untreated control (Ctrl) and cells treated with recombinant HOP (HOP). (B) Representative images of the migration assay of Parental (left) and shRNA-PrP2 (right) neurospheres 24 h after plating on laminin-1 (*n* = 4, **p* < 0.05). (PPTX 205 kb)

## Abbreviations

bFGF: Basic fibroblast growth factor
BSA: Bovine serum albumin
EGF: Epidermal growth factor
GBM: Glioblastoma
GSC: Glioblastoma stem-like cell
HOP: Hsp70/90 organizing protein
NSC: Neural stem cell
PBS: Phosphate-buffered saline
PrP^C^: Cellular prion protein
RT: Room temperature
