# Supplementary figures and images for: Engagement of cellular prion protein with the co-chaperone Hsp70/90 organizing protein regulates the proliferation of glioblastoma stem-like cells
Source: Stem Cell Res Ther. 2017 Apr 17;8:76. doi: 10.1186/s13287-017-0518-1 (PMC5392955; doi:10.1186/s13287-017-0518-1)

## Slide 1
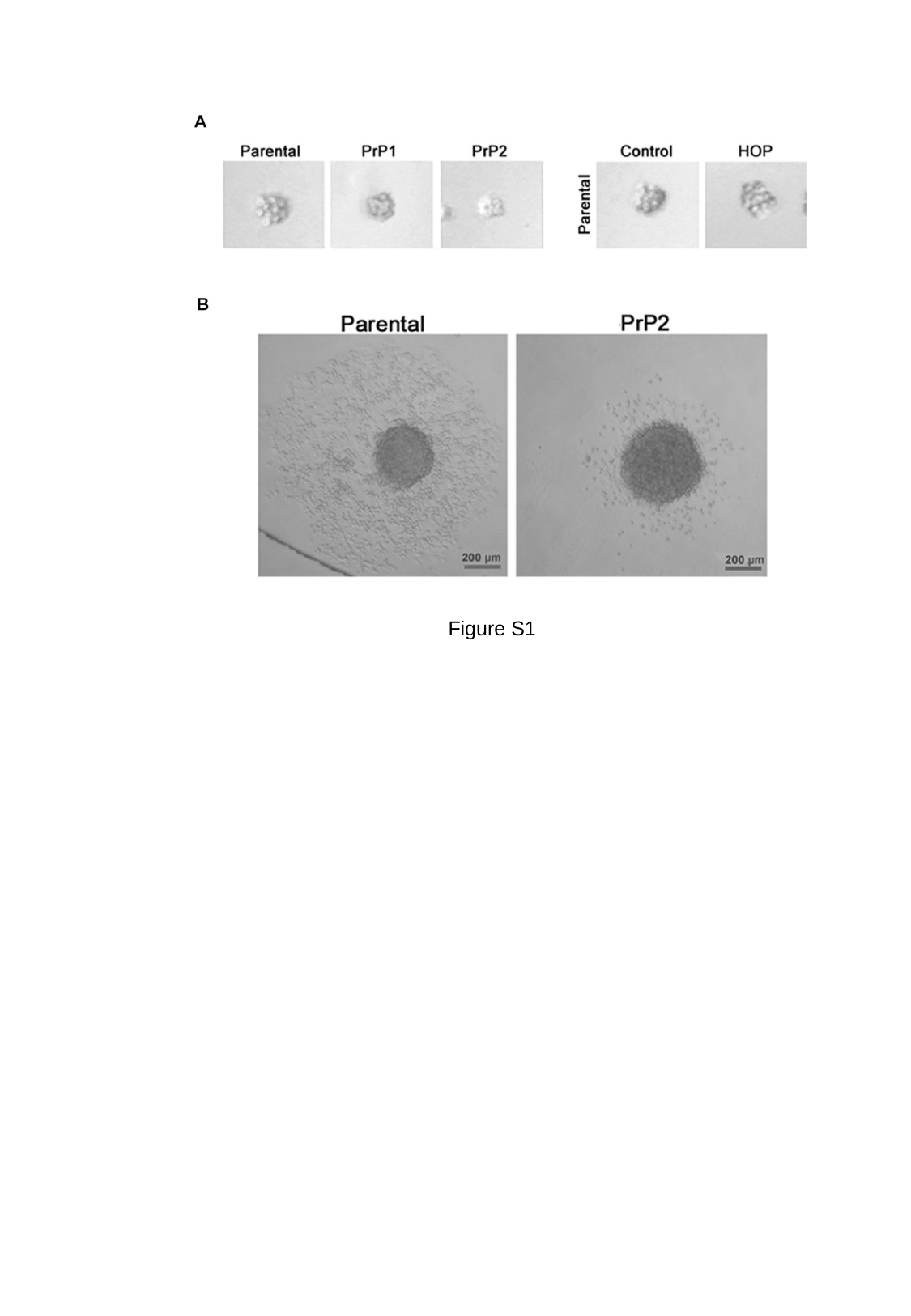

Figure S1

Supplement: Additional file 1: Figure S1. — Representative images of self-renewal and migration assays. (A) Representative image of self-renewal assay. Left panel: neurosphere growth in parental, shRNA-PrP1 (PrP1), and shRNA-PrP2 (PrP2) populations. Right panel: neurosphere size for the parental population untreated control (Ctrl) and cells treated with recombinant HOP (HOP). (B) Representative images of the migration assay of Parental (left) and shRNA-PrP2 (right) neurospheres 24 h after plating on laminin-1 (n = 4, *p < 0.05). (PPTX 205 kb) [file 13287_2017_518_MOESM1_ESM.pptx]
